# Using MRI derived patient-specific flow models and flow imaging for flow diverting stent rehearsal

**DOI:** 10.1186/1532-429X-15-S1-O56

**Published:** 2013-01-30

**Authors:** Gabriel Acevedo-Bolton, David Saloner

**Affiliations:** 1 Radiology, UCSF, San Francisco, CA, USA; 2 Radiology, VAMC, San Francisco, CA, USA

## Background

Flow diverting stents have recently been approved for use in treating vascular geometries not amenable to standard coiling or clipping. By using multiple stents, one inside another, the amount of blood passing through the stent walls can be controlled. Often, these stents must be deployed in aneurysms with tortuous vessels and complex geometries. The ability to rehearse stent deployment as well as to determine the number of stents required beforehand would be of great benefit to clinicians. In addition, the ability to non-invasively track the effects of these stents once deployed in vivo would be helpful in assessing whether the stent was having the intended effect. In our work, we show the utility of patient-specific flow models derived from CE-MRA to rehearse transcatheter stent placement and to assess the resulting flow conditions.

## Methods

Model construction: CE-MRA data were used to construct two patient-specific silicone models using a lost wax method. Each model was connected to a custom built flow pump, and gadolinium-doped water was pumped through. Stent placement: under physiologic flow conditions, interventionalists deployed the stents in the models under the guidance of fluoroscopy in an angiography suite. Flow imaging: 7D PC-MRI was performed on the flow model both prior to and after stent placement.

## Results

The flow models provided a very realistic test bed to rehearse the stent placement. In our models, the presence of the flow diverter stent was found to have a dramatic effect on the flow patterns within the aneurysm (Figure 2A and Figure 2B). Flow passing through the stent walls could be visualized by placing an emitter plane lateral to the stent location (Figure 2C).

**Figure 1:**
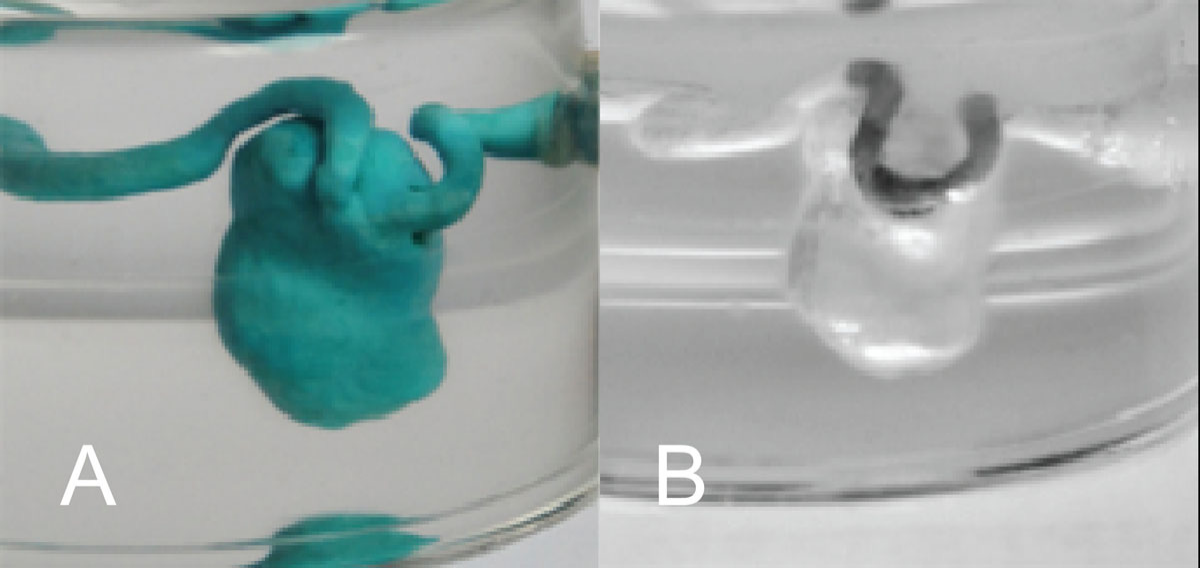
A) Wax model embedded in silicone B) model after wax removal and stent placement

**Figure 2:**
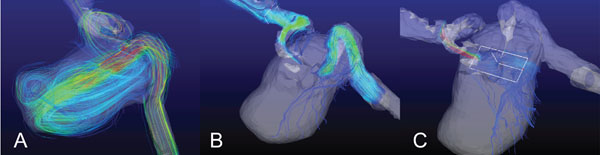
A) Streamlines in flow model of ICA aneurysm before stent B)After placement of stents and C)from an emitter plane offset from stent location.

## Conclusions

Patient specific flow models allowed interventionalists to rehearse stent deployment in complex geometries. These models in combination with MRI, allow for the assessment of the effect these stents have on flow patterns as well as an estimate of the total number of stents required for adequate flow reduction. Furthermore, the resulting artifacts from the stent is relatively small, as seen by the localized lack of streamlines in Figure 2b, thus allowing for longitudinal non-invasive monitoring of the aneurysm.

## Funding

NIH NS05873.

